# Evaluation of blood lead level in methamphetamine users in Tehran

**DOI:** 10.1186/s13011-017-0088-3

**Published:** 2017-02-22

**Authors:** Babak Mostafazadeh, Shahin Shadnia, Mohammad Ali Tavakkoli, Hamid Reza Khoddami Vishteh

**Affiliations:** 1 Department of Forensic Medicine, School of Medicine, Shahid Beheshti University of Medical Sciences, Loghman Hakim Hospital, Makhsoos St, South Karegar Ave, Tehran, Iran; 2 Department of Clinical Toxicology, School of Medicine, Shahid Beheshti University of Medical Sciences, Tehran, Iran; 3 Shahid Beheshti University of Medical Sciences, Tehran, Iran

**Keywords:** Crystal meth drug, Methamphetamine, Lead, Blood level

## Abstract

**Background:**

Given the increasing number of lead poisoning in opioids users and since no study has been conducted so far to review lead poisoning in methamphetamine (crystal) users, this study aimed to investigate blood lead level in methamphetamine addicts.

**Methods:**

This study was conducted on 20 patients with methamphetamine poisoning and their blood lead level was measured. The subjects were selected from among patients with a history of continuous use of methamphetamine, without a history of using opiates in the past 6 months confirmed by a negative urine tests, and without a history of heavy metal poisoning.

**Results:**

Of all, 18 patients were male and the mean age was 32 ± 10 years; 17 patients were abusing the drug via inhalation and three persons via oral administration. The mean blood lead level was 2.3 ± 1.1 μg/dL and poisoning was not observed in any of the cases. Blood lead level was not associated with age, sex, dosage, and route of administration.

**Conclusion:**

Although blood lead level was not at poisoning level in people who only used methamphetamine in Iran, due to the simultaneous use of other substances and because of non-specific symptoms, lead poisoning must be suspected in all cases of substances poisoning.

## Background

Lead is a heavy metal that is abundant in the environment and can cause acute and chronic poisoning. Items such as exhaust gas from cars, contaminated food, industrial waste water, and soil are the main sources of this type of pollution. Inhalation, oral, or dermal exposure to the mentioned sources of pollution can cause lead poisoning [1, 2]. Lead poisoning can cause non-specific protests such as abdominal pain, constipation, irritability, myalgia, anorexia, difficulty concentrating, decreased libido, headache etc. In addition, lead can cause irreversible neurological damage, kidney diseases, coronary artery diseases, and reproductive disorders. Nonspecific abdominal pain in this group of patients might be mistaken for acute abdomen, cholecystitis, and pancreatitis and may result in unnecessary abdominal examinations and even surgery [3].

Nowadays, due to increased level of safety in the workplace, the incidence of industrial lead poisoning has decreased greatly. However new forms of this type of poisoning has emerged. Since many years ago there have been a number of reports on lead poisoning caused by the use of substances such as opioids [4–7]. The existing evidence suggests an increasing number of substances abuse, especially opioids, in countries like Iran [8]; moreover, some studies in the Middle East, Iran, and other countries have reported the higher blood lead levels in substance users. On the other hand, an increasing number of studies have been conducted on opioids-related lead contamination and poisoning [3, 9–16]. According to a number of studies, some non-specific symptoms such as abdominal pain, neuropathy, and anemia in patients addicted to opioids have been improved via utilizing specific treatments for lead poisoning [9, 17–19]. Therefore, lead pollution in substances such as opioids is a new source of poisoning which is mainly due to adding lead or its combinations to illegal substances during the preparation process. However, existence of lead in the abused materials is a neglected fact that is sometimes even overlooked in scientific studies. For example, Kamangar et al suggest a link between opium use and cancer risk in their review article [20]. But according to the contamination of opium with lead which is confirmed in various studies in different countries, and especially Iran [13, 15], Mahmoodpoor et al said that this contamination could be a contributing factor for the high prevalence of cancer in opium users [21].

In addition to opioids and heroin [22, 23], there have been some reports on lead contamination and toxicity in other drugs, among which we may note marijuana [24] and methamphetamine [23, 25]. Nowadays, there is an increase in the number of amphetamine compounds in Iran. In Iran, crystal or Shisheh is a general name which is commonly used for some of these compounds [26, 27]. Recently, the transition from the traditional patterns of using opioids to methamphetamine use has changed in to a new health concern in Iran [28]. The increasing frequency of crystal is due to its illegal manufacturing, thus like other drugs, it is expected to observe cases of lead poisoning among people who use this type of drug. Although three studies were done on lead poisoning in the methamphetamine users [23, 25, 29], their date backed to more than 10 years ago and they were done on injecting methamphetamine, so this study was carried out to evaluate the blood lead level in inhalational and orally methamphetamine users.

## Methods

This cross-sectional study was conducted on patients with crystal (methamphetamine) poisoning who referred to Loghman Hakim poisoning center Tehran Iran, in 2014. Inclusion criteria were the followings: a history of continuous use of methamphetamine for more than 6 months, urine test positive for methamphetamine, not taking other opioids compounds (morphine derivatives) in the past 6 months, negative qualitative test of urine for drug compounds (morphine derivatives), and no history of heavy metal poisoning as confirmed by patients history. Everyday about ten patients with a history of methamphetamine use refer to emergency ward of Loghman hospital. However, in most cases, the duration of continuous use of methamphetamine is less than 6 months; in addition, most of the substance users simultaneously use other opioids compounds as well. Therefore, taking into consideration the inclusion criteria, during the 6 months of the study only 20 patients were diagnosed with sole methamphetamine poisoning and after obtaining informed consent from patients or their relatives, they were enrolled into the study. The study received an ethical approval from the Ethics Committee of Shahid Beheshti University of Medical Sciences.

A checklist was used to record patients’ demographic data including gender and age, the characteristics of methamphetamine use (duration of use, dosage, and route of administration), vital signs, and signs and symptoms at the time of the first visit. Then in accordance with the relevant guidelines, 5 ml blood sample was taken from each patient and sent to a laboratory to determine patients’ blood lead levels. Samples were tested via Atomic Abs method [30]. All the patients received supportive treatments and if tests were indicative of lead poisoning (greater than 25 μg/dL) the patients received specific treatments. Finally, patients’ outcomes were recorded.

SPSS for Windows version 22 was used for data analysis. Qualitative variables were described using frequency and percentage and quantitative variables were presented using mean and standard deviation. Moreover, independent *t* test was used to compare blood lead levels between sexes, age groups, and different consumption rates.

## Results

Of the 20 subjects, 18 patients (90%) were male and two patients (10%) were female. Patients’ age ranged from 20 to 54 years and their mean age (SD) was 32 ± 10 years. Of all, 17 patients (85%) were abusing the methamphetamine via inhalation and three persons (15%) were taking it orally. The range and mean (SD) dosage of methamphetamine used by patients, respectively, were 0.5–2 gr/day and 1 ± 0.5 gr/day in inhalation group and 1–10 gr/day and 4.7 ± 4.7 gr/day in oral administration group. The range and mean (SD) duration of methamphetamine abuse were 1–5 years and 2.7 ± 1.5 years, respectively. Table 1 presents the data on patients’ vital signs on admission and Table 2 presents the data on patients’ signs and symptoms on admission. The most common symptoms were hallucination and agitation. Only one patient (5%) required intubation.

**Table 1:** Vital signs of patients on admission

	Range	Mean (SD)
Temperature, centigrade	36–38	37 ± 0.5
Systolic blood pressure, mmHg	100–190	140 ± 24
Diastolic blood pressure, mmHg	60–140	86 ± 22
Heart rate	66–100	84 ± 11
Respiratory rate	14–26	18 ± 3

**Table 2:** Patients’ signs and symptoms on admission

	No (%)
Loss of consciousness	3 (15%)
Disorientation	5 (25%)
Hallucination	10 (50%)
Agitation	9 (45%)
Lethargy	1 (5%)
Seizure	2 (10%)
Hypertension	2 (10%)

The range and mean (SD) of blood lead levels in patients were 0.6–4.6 μg/dL and 2.3 ± 1.1 μg/dL, respectively, and lead poisoning was not observed in any of the cases. Blood lead level was not associated with age, sex, dosage, and route of administration (Table 3). All patients received supportive therapy. Finally, one patient (5%) was hospitalized in ICU, 14 patients (70%) were hospitalized in general ward, and 5 patients (25%) were discharged.

**Table 3:** Relation of lead level and patients’ characteristics

		Mean (SD)	*P* Value*
Sex	Male	2.2 ± 1.1	0.313
	Female	2.9 ± 0.2	
Age group	<= 30 year	2.4 ± 1.1	0.543
	>30 year	2.0 ± 1.0	
Administration route	Inhalation	2.3 ± 1.0	0.711
	Oral	2.0 ± 1.0	
Dosage	<= 0.5 gr/day	2.8 ± 1.2	0.128
	>0.5 gr/day	2.1 ± 0.8	

* Mann-Whitney U tests

## Discussion

According to the results of this study, in the 20 patients with sole methamphetamine poisoning, the mean (SD) blood lead level was 2.3 ± 1.1 μg/dL, thus no case of lead poisoning was observed in the subjects. Moreover, blood lead level was not associated with age, sex, dosage, and route of administration.

Despite extensive searches, we only found three studies about lead poisoning in methamphetamine users. In Allcott et al.’s study in 1987, lead poisoning was reported in two patients who had been injecting illegal methamphetamine. According to their study, acute lead poisoning can present with hepatitis, nephritis, and encephalopathy [25]. In another study in 1989, lead poisoning is reported in 12 patients who had been injecting methamphetamine. At the same time, the tests on illegal methamphetamine showed that up to 60% of the weight of the tested drug was composed of lead [29]. In another study in 1996, Norton et al. studied blood lead levels in 92 injecting methamphetamine users and compared them with blood lead levels in 53 substance users who were injecting other types of substances. As the results showed, blood lead level was 6.21 μg/dL in methamphetamine group and 7.25 μg/dL in non-methamphetamine users and there was no significant difference between the two groups [23]. They also stated that previous reports which had suggested lead poisoning in this group of patients might have been due to accidental contamination of methamphetamine with lead [23].

Our study is the fourth study measuring blood lead levels in methamphetamine users. Various combinations of methamphetamine is used in Iran, among which crystal (Shisheh, as common name in Iran) is the most common form of handmade methamphetamine. Methamphetamine is mainly used via inhalation; however, other methods such as oral administration, smoking, and injection are also common [31]. A large number of methamphetamine users simultaneously use other types of opioids as well. Hence, in our study only 20 cases of poisoning due to consumption of pure methamphetamine were found within the 6 months of the study. However, the mean blood lead level was 2.3 μg/dL which was much lower than the levels of poisoning. In addition, no significant relationship was observed between blood lead level and age, sex, dosage, and route of administration. So we can say that although methamphetamine is illegally produced similar to other opioids in Iran, apparently it does not have problems related to lead poisoning which have been observed in opioids users. Nevertheless, as the symptoms of lead poisoning are non-specific and since many substance users in Iran use several types of substances such as opioids together with methamphetamine, lead poisoning must be suspected in all cases of drug poisoning and, if necessary, proper diagnostic procedures such as blood lead levels tests must be performed to reach a definitive diagnosis and start treatment subsequently.

## Conclusion

According to the findings of this study, no case of lead poisoning was found in the patients with sole methamphetamine use in our country. However, due to illegally production of these substances, the simultaneous use of other substances, and non-specific symptoms, lead poisoning must be suspected in all cases of substance poisoning and, if necessary, proper measures must be taken to diagnose and treat the patients.
